# A Nanotechnological Approach to Exploit and Enhance the Bioactivity of an Extract from *Onopordum illyricum* L. Leaves

**DOI:** 10.3390/plants12071453

**Published:** 2023-03-26

**Authors:** Carla Caddeo, Carlo Ignazio Giovanni Tuberoso, Sonia Floris, Valentina Masala, Cinzia Sanna, Francesca Pintus

**Affiliations:** 1 Department of Life and Environmental Sciences, University of Cagliari, SS 554—Bivio per Sestu, Monserrato, 09042 Cagliari, Italy; 2 Department of Life and Environmental Sciences, University of Cagliari, Via S. Ignazio da Laconi 13, 09123 Cagliari, Italy

**Keywords:** *Onopordum illyricum*, phenolic composition, LC-ESI-QTOF MS/MS, phospholipid vesicles, DPPH/FRAP assays, anti-ROS activity, cell culture

## Abstract

Plant-derived products have been used for preventive and curative purposes from the ancient era to the present day. Several studies have demonstrated the efficacy of either multicomponent-based extracts, enriched fractions, or isolated bioactives. However, they often display low solubility and bioavailability, chemical instability, poor absorption, and even toxicity, which restrict application in therapy. The use of drug delivery systems, especially nanocarriers, can overcome these physicochemical and pharmacokinetic limitations. In this study, an extract from *Onopordum illyricum* leaves was produced by maceration in 80% ethanol, characterized by liquid chromatography coupled to mass spectrometry, and formulated in phospholipid vesicles with the aim of exploiting and possibly enhancing its bioactivity for skin delivery. The results showed that phenolic compounds were abundantly present in the extract, especially hydroxycinnamic acid and flavonol derivatives. The extract-loaded vesicles showed small size (<100 nm), high entrapment efficiency (even >90% for most phenolic compounds), and good long-term stability. Moreover, the extract-loaded vesicles exhibited remarkable antioxidant activity, as demonstrated by colorimetric assays and by enhanced reduction of intracellular reactive oxygen species (ROS) levels in cultured skin cells. Hence, our findings support the key role of nanotechnological approaches to promote the potential of plant extracts and strengthen their application in therapy.

## 1. Introduction

*Onopordum illyricum* L. is a wild thistle belonging to Asteraceae family, widespread in the Mediterranean basin. It is a biennial herb, known as Illyrian thistle or Illyrian cottonthistle (in Italy it is called “Onopordo Maggiore”), which exceeds two meters in maximum height. It produces spiny leaves, up to 50 cm long, and inflorescences made of large purple flower heads.

It is traditionally used for food and therapeutic purposes. In Sardinia (Italy), its young scapes and capitula are eaten raw in salad [1]. The whole plant is used in decoction as cough sedative, digestive, and for biliary disease, while flower heads are used in decoction or infusion as antipyretic to treat malarial fever, and for washing exanthematous skin [2].

*O. illyricum* was found to be rich in sesquiterpene lactones [3,4,5,6,7,8], caffeoylquinic acid derivatives, and flavonoids [8,9]. Out of the compounds isolated, onopordopicrin and vernomelitensin, as well as 3,5 dicaffeoylquinic acid, exhibited a strong antinflammatory activity, inhibiting interleukin-8 (IL-8) release, pro-inflammatory transcription factors NF-κB, and signal transducer and activator of transcription 3 STAT3, and also activating the transcription factor Nrf2 [7,9]. Furthermore, 1,5-dicaffeoylquinic acid and luteolin showed anti-HIV-1 activity, inhibiting both reverse transcriptase and integrase enzymes [8].

In the last decades, there has been an increasing interest in the search for novel pharmacophores from plants because of their chemical diversity and versatility not matched by synthetic chemistry libraries. Various biological effects have been described for plant-derived compounds. The latter, in the form of either isolated molecules or extracts, might find applications in foods, cosmetics, and pharmaceuticals, but their direct incorporation into viable products presents certain difficulties [10]. Plant-derived products are often prone to degradation and possess poor aqueous solubility and limited permeability that hinder their dissolution and absorption. In this context, nanotechnologies can be a valuable strategy to overcome these limitations. Phospholipid vesicles are being successfully used to load plant extracts and deliver them to a number of targets via different administration routes, and several studies have demonstrated their greater therapeutic efficacy compared with conventional approaches [11,12]. Nanocarriers have been proved to be effective in resolving critical issues of natural compounds, such as poor bioavailability, low stability in physiological environment, low site-specific distribution, and side effects due to multi-targeting [13,14].

In this study, the possibility to exploit and even enhance the bioactivity of an *O. illyricum* extract by using nanotechnologies was investigated. Particularly, a potential topical application of vesicle formulations for the treatment of oxidative stress-related skin disorders is proposed. An extract from *O. illyricum* leaves was produced and characterized by identifying phenolic compounds commonly known for their biological importance. Two types of phospholipid vesicles (i.e., liposomes and Penetration Enhancer-containing Vesicles, PEVs) were prepared to improve the extract’s bioavailability and applicability on the skin. The vesicles were characterized by size distribution, surface charge, stability, and entrapment efficiency. In addition, their antioxidant power was evaluated via colorimetric assays (DPPH and FRAP) and in cultured skin cells.

## 2. Results

### 2.1. Quali-Quantitative Determination of Phenolic Compounds in O. illyricum Extract

The extract obtained from *O. illyricum* leaves was qualitatively analyzed by (HR) LC-ESI-QTOF MS/MS in negative ion mode, and targeted phenolic compounds were quantified by LC-DAD analysis.

**Figure 1:**
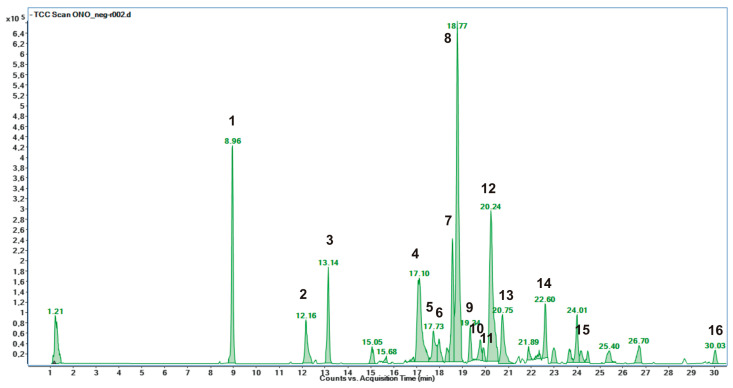
(HR) LC-ESI-QTOF MS/MS total compound chromatogram (TCC) of *O. illyricum* extract acquired in negative ion mode. Peak identification is given in Table 1.

The negative LC-MS profile highlighted the presence of a large group of compounds corresponding to the deprotonated molecular ions of different phenolic derivatives, mainly hydroxycinnamic acid and flavonoid derivatives (Figure 1). Individual components were identified by comparison of their *m*/*z* values in the total compound chromatogram (TCC) profile with those described in the literature (see References in Table 1). Moreover, by comparing experimental MS/MS spectra with fragmentation patterns reported in the literature for the same analytes or with the fragmentation patterns and spectra reported in a public repository of mass spectral data [15,16,17], 16 compounds were identified. Table 1 reports the identified compounds, listed according to their retention times (Rt), the UV maximum absorbance (λmax), the chemical formula derived by accurate mass measurement, MS/MS results, the references used for identification, and the identification confidence levels [18].

LC-DAD analysis supported the investigation of the phenolic fraction. Indeed, based on the evaluation of the typical UV-vis spectrum acquired between 200 and 600 nm, the most representative wavelength of maximum absorbance (λmax) and through the comparison with the literature data, it was possible to attribute the compounds to specific polyphenolic classes and identify with certainty some molecules (Table 1). Finally, polyphenols were quantified at 360 nm for flavonols and at 313 nm for hydroxycinnamic acids.

The most representative compounds belong to the class of hydroxycinnamates. The reference nomenclature for caffeoylquinic acid derivatives used in this work is the IUPAC one [23]. Compounds **1**–**3**, **5**–**8**, **10**–**12** and **14** were identified as hydroxycinnamic derivatives. Peak **1** was identified as a mono-caffeoylquinic acid, due to the [M−H]^−^ *m*/*z* 353.0884 and a fragment at *m*/*z* 191.0559 (quinic acid unit) [4,20]. By comparison with a pure standard, the peak was attributed to 5-*O*-caffeoylquinic acid, well known as chlorogenic acid. Peaks **2**, **5**, **6**, **7**, **8** and **11** were identified as dicaffeoylquinic acids due to the [M−H]^−^ *m*/*z* 515.1197 and the fragments at *m*/*z* 191.0560, 353.0884 (chlorogenic acid unit) and 179.0339 (caffeic acid unit) [4,20]. Again, by comparison with pure standards, the peaks **2**, **5**, **6** and **11** were attributed to 1,3-dicaffeoylquinic acid (cynarin), 3,4-dicaffeoylquinic acid (isochlorogenic acid B), 3,5-dicaffeoylquinic acid (isochlorogenic acid A) and 4,5-dicaffeoylquinic acid (isochlorogenic acid C), respectively. Peak **8** was tentatively attributed to 1,5-dicaffeoylquinic acid due to the [M−H]^−^ *m*/*z* 515.1197 and fragments at *m*/*z* 353.0863, 191.0562, and 179.0339, and by comparison with literature data [4,8,9]. Compound **3** was tentatively identified as feruloylquinic acid due to the [M−H]^−^ *m*/*z* 367.1038 and two fragments at *m*/*z* 191.0560 and 193.0482 (loss of ferulic acid unit) [19]. Peak **14** was tentatively attributed to a feruloylcaffeoylquinic acid due to the [M−H]^−^ *m*/*z* 529.1354 and the fragments at *m*/*z* 367.1038 (feruloylquinic acid unit), 353.0884 and 191.0560 [19]. Compounds **10** and **12** were tentatively attributed to dicaffeoylsuccinoylquinic acids due to the [M−H]^−^ *m*/*z* 615.1361 [4,9,20]. Besides being the most represented compounds, hydroxycinnamic acids were also the most abundant phenolic compounds in *O. illyricum* (9.59 ± 0.07 mg/g dm; Table 2). Dicaffeoylquinic acids accounted for 80% of all hydroxycinnamic acids, and 1,5-di-*O*-caffeoylquinic acid was the most abundant (3.40 ± 0.07 mg/g dm).

Compounds **4**, **9**, **13**, **15** and **16** were attributed to the phenolic class of flavonols by the study of the UV-vis spectra through LC/DAD and the interpretation of mass spectra and MS/MS fragments. Compound **4** was identified as luteolin glucuronide due to the [M−H]^−^ *m*/*z* 461.3725 and a fragment at *m*/*z* 285.0404, corresponding to a residue of luteolin aglycon. Neutral loss of 176 allowed us to identify the glucuronide unit [21]. A similar [M−H]^−^ *m*/*z* was found in a previous study on *O. illyricum* by Verotta et al. [4], and was attributed to a kaempferide glucoside, but the UV-vis spectrum is more consistent with a luteolin derivative than a kaempferide one. Peak **9** was tentatively attributed to apigenin rutinoside (isorhoifolin) due to the [M−H]^−^ *m*/*z* 577.1564 and a fragment at *m*/*z* 269.0452, as well as comparison with literature data [4]. Compound **13** was tentatively attributed to hispidulin glucuronide due to the [M−H]^−^ *m*/*z* 475.0880 and fragments at *m*/*z* 299.0535 and 284.0334. Compounds **15** and **16** were attributed to luteolin and hispidulin due to their [M−H]^−^ *m*/*z* 285.04 and 299.0558, respectively, and the interpretation of UV-vis spectra, with the confirmation of literature data [4,8]. Total flavonols amount accounted for 7.32 ± 0.05 mg/g dm, and luteolin glucuronide was the most abundant flavonol compound (4.37 ± 0.00, mg/g dm), followed by hispidulin and luteolin (Table 2).

### 2.2. Vesicle Characterization

Two vesicle formulations, namely liposomes and propylene glycol-Penetration Enhancer-containing Vesicles (PG-PEVs), were developed and characterized in terms of mean diameter, polydispersity, and zeta potential. To evaluate the extract’s impact on the vesicles’ characteristics, the liposomes and PG-PEVs with *O. illyricum* extract were compared with the empty liposomes and empty PG-PEVs. The light-scattering results, as reported in Table 3, showed that both the empty liposomes and the empty PG-PEVs were around 80 nm in diameter, slightly polydispersed (PI > 0.3), and negatively charged (approximately −14 mV). The extract’s loading significantly increased the liposomes’ mean diameter, although they remained small (96 nm), whereas it had no effect on the PG-PEVs. Furthermore, the extract’s loading significantly decreased the polydispersity index and zeta potential values (Table 3). 

The stability of the vesicle formulations was evaluated by monitoring the mean diameter, the polydispersity index, and the zeta potential during storage. No signs of significant alterations were detected in the formulations containing the extract, since the vesicles remained small and monodispersed (Table 4). The empty vesicles were less stable, as indicated by increased mean diameter and polydispersity index values (90 nm and 0.4, respectively; Table 4).

The entrapment efficiency (EE) of the two nanoformulations was calculated based on the amount of 13 targeted phenolic compounds identified in the *O. illyricum* extract and detected in the dialyzed and non-dialyzed vesicle dispersions (Table 5). In general, liposomes provided greater entrapment efficiency values than PG-PEVs, even above 90% for most hydroxycinnamic acids and the flavonols.

### 2.3. Antioxidant Assays

The antioxidant activity of the *O. illyricum* extract was estimated as a function of its radical scavenging and ferric-reducing abilities (Table 6). The extract solution scavenged the DPPH radical completely (AA 94%), corresponding to approximately 670 μg/mL of Trolox equivalents. Given the presence of phosphatidylcholine, empty vesicles displayed a slight antioxidant activity (AA 39%). The level of antioxidant activity for the *O. illyricum* vesicles was similar to that of the extract solution: AA 99% and approximately 700 μg/mL of Trolox equivalents, without statistical differences (Table 6).

The results of the FRAP assay showed that the extract solution had a strong reducing power (approximately 450 μg/mL of ferrous equivalents; Table 6). Additionally, in this case, the empty vesicles showed a slight reducing power (190 μg/mL of ferrous equivalents), which contributed to the activity detected for the *O. illyricum* vesicles, which was above 550 μg/mL of ferrous equivalents (*p* < 0.01 vs. the extract solution).

These findings confirm that the antioxidant activity of the *O. illyricum* extract was retained, or even enhanced, in the vesicle formulations.

### 2.4. Cell Viability and Anti-ROS Activity

**Figure 2:**
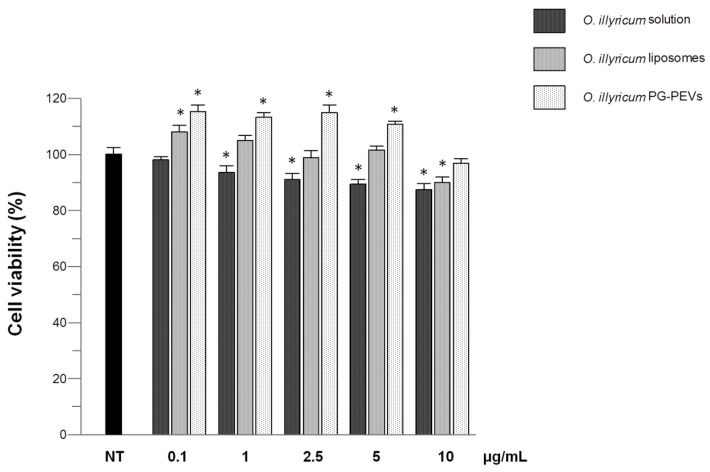
Effect of *O. illyricum* solution and *O. illyricum* liposomes/PG-PEVs on HaCaT cell viability. After 24 h of incubation with samples at different concentrations (0.1–10 µg/mL), cell viability was determined by the MTT assay. Asterisks indicate the statistical differences (*p* < 0.01) vs. non-treated control cells (NT). At each concentration, the three samples were also statistically different (*p* < 0.001) from each other, with the exception of *O. illyricum* solution vs. *O. illyricum* liposomes at 10 µg/mL.

The effects of *O. illyricum* solution (2 mg/mL in DMSO) and *O. illyricum* liposomes/PG-PEVs were examined on cell viability, after proper dilution of samples to reach the concentrations of 0.1, 1, 2.5, 5 and 10 µg/mL. To this end, HaCaT cells were treated with the *O. illyricum* samples for 24 h and the viability was investigated using the MTT test. The results indicated that none of the samples were cytotoxic in HaCaT cells. A slight decrease in viability was observed in the cells exposed to the *O. illyricum* solution, reaching the lower value (87%) at the higher concentration (10 μg/mL) (Figure 2). On the other hand, an increase in viability was observed in the cells exposed to the *O. illyricum* nanoformulations in the 0.1–5 μg/mL concentration range.

**Figure 3:**
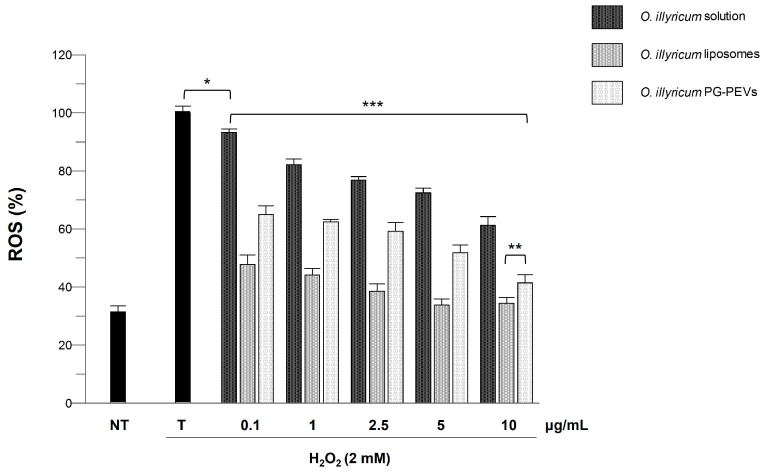
Inhibition of H_2_O_2_-induced ROS generation (1 h-incubation with 2 mM H_2_O_2_) by *O. illyricum* solution and *O. illyricum* liposomes/PG-PEVs in HaCaT cells. NT, non-treated cells; T, cells treated with H_2_O_2_ only. *** Values statistically different (*p* < 0.0001) from each other and from cells treated with H_2_O_2_ only (T), with the exceptions of 0.1 μg/mL *O. illyricum* solution vs. cells treated with H_2_O_2_ only (* *p* < 0.05) and 10 μg/mL *O. illyricum* liposomes vs. PG-PEVs (** *p* < 0.005).

In addition, ROS levels in cells were evaluated before and after H_2_O_2_-induced oxidative stress and upon treatment with *O. illyricum* solution or *O. illyricum* liposomes/PG-PEVs. H_2_O_2_-generated ROS in the cytoplasm oxidate DCFH to fluorescent DCF, whose levels were quantified spectrophotometrically. As shown in Figure 3, the incubation of the HaCaT cells with H_2_O_2_ significantly increased ROS levels (vs non-treated cells). Treatment with *O. illyricum* solution and *O. illyricum* liposomes/PG-PEVs prevented ROS production in a dose-dependent manner. In particular, the greatest intracellular ROS inhibition was provided by *O. illyricum* liposomes, followed by *O. illyricum* PG-PEVs, and finally *O. illyricum* solution. Considering each concentration, both vesicle formulations were statistically more efficient (*p* < 0.0001) than the solution. Thus, these results demonstrate that the two vesicle formulations enhanced the anti-ROS activity of the extract.

## 3. Discussion

The phytochemical characterization of *O. illyricum* extract displayed a rich phenolic fraction in accordance with previous studies [4,8,9]. The most representative compounds are widely present in other plants belonging to the Asteraceae family. Luteolin-7-glucuronide was previously found in *Achillea millefolium* [24] and *Cynara scolymus* [25], and hispidulin glucuronide was previously identified in *Centaurea castriferri* [22]. Other compounds were described for *O. illyricum*, mainly belonging to the group of sesquiterpene lactones and neolignan derivatives [3,4,5,6,7,8].

*O. illyricum* has been previously investigated for its antinflammatory, antiviral, and antifeedant properties [6,7,8,9], but the antioxidant activity was never investigated before, neither by using colorimetric assays nor in skin cells. We observed a strong antioxidant activity, both in cell-free systems and in cultured skin cells, that could be correlated to the high phenolic content of the extract. Our results are consistent with the literature, since a linear correlation between the antioxidant activity and the total phenolic content has been frequently described [26,27]. Among the specialized metabolites identified in our study, particularly 1,5-dicaffeoylquinic acid has been related to a strong antioxidant activity [28]. Moreover, out of the flavonols detected, luteolin has been previously reported for its antioxidant capacity [29], while hispidulin showed contrasting results, being not active in cell-free systems but effective in the protection against erythrocyte membrane lipid peroxidation [30].

Furthermore, to the best of our knowledge, this is the first study that reports on the exploitation of an *O. illyricum* extract by using nanotechnologies to favor its application on the skin for therapeutic purposes. In our study, liposomes and PG-PEVs were developed to load the extract. The obtained results point to a strong interaction between the phospholipid and the extract, rather than the penetration enhancer PG, and to a consequent modification of the arrangement of the bilayer leading to nanosized and more homogeneous vesicles. The good storage stability and the high entrapment efficiency of key phenolic compounds highlighted the vesicles’ potential as vehicles for the *O. illyricum* extract. These findings are in agreement with other studies reporting that phospholipid vesicles provided high entrapment efficiency, narrow polydispersity, and good stability of plant extracts [31,32,33].

The retention and even enhancement of the antioxidant activity of the *O. illyricum* extract after the nanoformulation process was demonstrated. The results obtained in keratinocytes showed that the nanoformulations, liposomes especially, protected the cells from chemically generated ROS more efficiently than the free extract. A 35% reduction of intracellular ROS levels was measured in cells exposed to the extract solution at the higher concentration (10 μg/mL). The same extent of reduction was achieved with extract-loaded PG-PEVs at a 100-times lower concentration (0.1 μg/mL). At this concentration, extract-loaded liposomes provided a 50% reduction, and at a 5 μg/mL concentration, ROS levels were comparable with those recorded in non-treated cells. A similar enhancement of the antioxidant activity of a plant extract was demonstrated by De Luca et al. [34]. The authors reported that basal intracellular ROS levels were restored in stressed skin cells when *Myrtus communis* L. extract liposomes were used. Hence, the nanotechnologies, which had the primary task of allowing the development of formulations suitable for skin application, were proved to represent a valuable approach for exploiting and potentiating the bioactivity of *O. illyricum* extract.

## 4. Materials and Methods

### 4.1. Chemicals

Phospholipon90G (P90G; phosphatidylcholine content ≥ 94%) was purchased from Lipoid GmbH (Ludwigshafen, Germany). Propylene glycol (PG) were purchased from Galeno Srl (Comeana, Prato, Italy). LC-MS grade acetonitrile, formic acid, methanol, 85% w/w phosphoric acid, 2,2-diphenyl-1-picrylhydrazyl (DPPH), 6-hydroxy-2,5,7,8-tetramethylchroman-2-carboxylic acid (Trolox), 2,4,6-tris(pyridin-2-yl)-1,3,5-triazine (TPTZ), 3-(4,5-dimethylthiazol-2-yl)-2,5-diphenyltetrazolium bromide (MTT), 2′,7′-dichlorofluorescein diacetate (DCFH-DA), dimethyl sulphoxide (DMSO), and all the other reagents, if not otherwise specified, were purchased from Sigma-Aldrich/Merck (Milan, Italy). Standards of 5-*O*-caffeoylquinic (chlorogenic acid), 1,3-dicaffeoylquinic acid (cynarin), 3,5-dicaffeoylquinic acid (isochlorogenic acid A), 3,4-dicaffeoylquinic acid (isochlorogenic acid B), 4,5-dicaffeoylquinic acid (isochlorogenic acid C), ferulic acid, apigenin, hispidulin, and luteolin were purchased from Extrasynthese (Genay, France) and TransMIT (Giessen, Germany). Ultrapure water (18 MΩ·cm) was obtained with a Milli-Q Advantage A10 System apparatus (Millipore, Milan, Italy).

### 4.2. Plant Material

*O. illyricum* leaves were collected in Gairo Taquisara (Sardinia, Italy) in June 2021. The sampling was carried out at the flowering stage of the plant. The leaves were randomly harvested from 10 individuals of the same population. The species was botanically identified by Cinzia Sanna, and a voucher specimen (CAG 798/V1) was deposited in the General Herbarium of the Department of Life and Environment Sciences, University of Cagliari. Plant material was dried in a ventilated stove at 40 °C to constant weight.

### 4.3. Extract Preparation

Dried leaves of *O. illyricum* (40 g) were ground in an electric grinder and the obtained powder was extracted with 80% ethanol three times, at room temperature for 24 h. The obtained extracts were joined, filtered, and concentrated under reduced pressure to evaporate the alcohol. The resulting aqueous phase was first frozen to −80 °C and then freeze-dried, affording 4.2 g of crude extract that was stored at −20 °C until use.

### 4.4. High-Resolution LC-ESI-QTOF-MS-MS Analysis

Qualitative investigation of the O. illyricum extract was performed by an ion mobility QTOF LC-MS system using a 1290 Infinity II UPLC equipped with an autosampler (G7167B), a quat pump (G7120A), a column comp (G7116B), and 6560 IM-QTOF (Agilent Technologies Inc., Santa Clara, CA, USA). Overall instrument performances were tested before analysis using an Agilent tuning solution mix (G1969-85000), and during the analysis, two reference masses at *m*/*z* 112.9855 and *m*/*z* 966.0007 were continuously infused to the system for constant mass correction. The electrospray ionization (ESI) source in negative ion mode was used to perform all the experiments and the optimized source parameters were the follows: drying gas at 300 °C with a flow of 5 L/min, sheath gas at 250 °C with at 12 L/min, nebulizer at 45 psi, capillary voltage set to 3500 V with a nozzle voltage of 500 V. Automatic acquisition MS/MS experiments were carried out applying a formula to calculate the collision energy by linear interpolation, calculated according to the following equation: collision energy = [slope (5) × *m*/*z* of precursor mass]/100 + Offset (2). The mass spectra were acquired by full-range acquisition, covering the m/z range of 40–1300.

Chromatographic separation was performed on a Kinetex EVO C18 column (150 × 2.1 mm, 1.7 µm 100 Å; Phenomenex, Castel Maggiore, Bologna, Italy) maintained at 55 ± 1 °C. The mobile phase consisted of a combination of solvent A (0.1% formic acid) and solvent B (acetonitrile + 0.1% formic acid) at a flow rate of 0.3 mL/min. The gradient elution was as follows: 0–20 min (99–80% A), 20–35 min (80–70% A), 35–40 min (70–1% A), 40–45 min (1–1% A), 45–46 min (1–99% A) and 46–50 min (99–99% A). The injection volume was 4 µL.

Data acquisition and processing were done using Agilent MassHunter Workstation Acquisition software v. B.09.00 (Agilent Technologies). ESI/QTOF MS data were then analyzed using the molecular feature extraction algorithm of the MassHunter Workstation Qualitative Analysis software v. 10.0 (Agilent Technologies). The tentative identification and analysis of LC-MS/MS data for the metabolites were carried out using the MassHunter METLIN metabolite PCDLdatabase v. B.08.00 (Agilent Technologies) and Sirius^®^ software v. 4.7.4 to predict fragmentation and molecular formulae [16,17].

### 4.5. HPLC-DAD Analysis

Quantitative analysis of targeted phenolic compounds was carried out using a modified HPLC-DAD method, as described by De Luca et al. [34] using an Agilent 1260 Infinity II HPLC system (Agilent Technologies) fitted with a pump module G7111A, an autosampler module G7129A, a thermostatted HPLC column compartment G7116A (30 ± 1 °C), and an Agilent G4212B photodiode array detector. The separation was obtained with a Kinetex EVO C18 column (150 × 4.60 mm, 2.6 μm, Phenomenex, Casalecchio di Reno, Bologna, Italy) using 0.22 M phosphoric acid (solvent A) and acetonitrile (solvent B) as the mobile phase, at a constant flow rate of 0.8 mL/min. The gradient (*v*/*v*) was generated decreasing from 100% solvent A to 80% in 20 min, to 70% in 35 min, to 0% in 45 min, and then remaining stable up to 50 min; finally, the gradient achieved 100% solvent A and remained stable for 5 min before following injection. The injection volume was 10 μL. The chromatograms and spectra, acquired in the wavelength range 200–600 nm, were elaborated with an OpenLab CDS software v. 2.5 (Agilent Technologies). Stock standard solutions were prepared in methanol and working standard solutions were prepared in 0.22 M phosphoric acid. The calibration curves for commercial standards (see Section 4.1) were plotted with the method of the external standard, correlating the peak area with the concentration by means of the least-squares method, with a coefficient of determination (r^2^) > 0.999 in the range of 1–200 mg/L for all the compounds. Quantification of phenolic compounds for which no pure standards were available was obtained by comparison with the most similar calibration curve. Compounds **3** and **14** were dosed as ferulic acid equivalents, compounds **8**, **10**, and **12** as 1,3-di-O-caffeoylquinic acid equivalents, compound **4** as luteolin equivalents, compound **9** as apigenin equivalents, and compound **13** as hispidulin equivalents. Phenolic compound amounts were expressed as mg/g dm (extract dry mass). For the LC-DAD analysis, the O. illyricum extract was dissolved in methanol (2 mg/mL) and diluted 1:50 *v*/*v* with methanol, as well as the nanoformulations. The solutions were filtered with 0.22 μm CA syringe filter before injection.

### 4.6. Vesicle Preparation and Characterization

Liposomes and Penetration Enhancer-containing Vesicles (PEVs) were developed. The liposomes were prepared by dispersing P90G and the *O. illyricum* extract in water (Table 7) and sonicating with a Soniprep 150 plus disintegrator (MSE Crowley, London, UK; 11 cycles of 5 s on/2 s off + 9 cycles of 2 s on/2 s off; 13 µm of probe amplitude).

For the preparation of PEVs, the penetration enhancer propylene glycol (PG) was included in the formulation. PG-PEVs were prepared according to the same protocol used for liposomes, but with the addition of 10% *v*/*v* PG. For a proper comparison, empty vesicles were prepared according to the same procedure as extract-loaded vesicles, but without the extract (Table 7).

The mean diameter, polydispersity index (PI), and zeta potential of the Et-PEVs were determined by dynamic and electrophoretic light scattering using a Zetasizer nano-ZS (Malvern Panalytical, Worcestershire, UK). The samples (*n* > 10) were diluted with water (1:100) and analyzed at 25 °C.

The above three parameters were monitored for 60 days to assess the long-term stability of the nanoformulations, stored at 4 °C.

The non-incorporated extract components were removed from the liposomes and PG-PEVs dispersions through dialysis. A 1 mL sample was loaded into Spectra/Por^®^ tubing (12,000–14,000 Da MWCO; Spectrum, Breda, The Netherlands) and kept in water (2 L), under gentle stirring. After 2 h, dialyzed vesicles were disrupted by diluting (1:50 *v*/*v*) with methanol, as well as non-dialyzed vesicles, and analyzed by LC–DAD to determine the amounts of targeted phenolic compounds (see Section 4.4). The entrapment efficiency (EE) was calculated as the percentage of the hydroxycinnamic acids and flavonols detected in dialyzed vs. non-dialyzed liposomes and PG-PEVs.

### 4.7. Antioxidant Assays

The DPPH assay was carried out to evaluate the antioxidant activity of *O. illyricum* extract (2 mg/mL) in methanol solution or in liposomes/PG-PEVs. Empty vesicles were also tested to evaluate the activity of the nanocarriers. Each sample (40 µL) was added to a DPPH methanolic solution (25 µM; 2 mL) and incubated at room temperature in the dark for 30 min. The discoloration of the DPPH solution, which occurs as a function of the antioxidant power and the concentration of a sample, leads to a decrease in absorbance (*A*) at 517 nm. The antioxidant activity (*AA*) of the samples was calculated according to Equation (1):

(1) $$AA=\left(\frac{A_{DPPH}-A_{sample}}{A_{DPPH}}\right)\times 100$$

The results were expressed also as Trolox equivalents (TEs). The TE values (µg TE/mL solution) were calculated by using a calibration curve (Trolox concentration range: 50–750 µg/mL).

The antioxidant activity of *O. illyricum* extract (2 mg/mL), either in methanol solution or in liposomes/PG-PEVs, and empty vesicles was assessed also by the FRAP (ferric reducing antioxidant power) assay. The test is based on the reduction of Fe^3+^-TPTZ to Fe^2+^-TPTZ that causes an increase in absorbance. A total of 40 µL of each sample was mixed with 2 mL of the TPTZ–ferric solution. After 4 min of incubation at room temperature in the dark, the absorbance was read at 593 nm. The results, expressed as ferrous equivalents (FE; µg Fe^2+^ equivalents/mL solution), were calculated by using a calibration curve (FeSO_4_ concentration range: 13.9−1112 µg/mL).

### 4.8. Cell Viability

Human keratinocytes (HaCaT; CLS–Cell Lines Service, Eppelheim, Germany) were cultured under standard conditions (5% CO_2_, 95% relative humidity, and 37 °C) in Dulbecco’s Modified Eagle’s Medium plus 1% penicillin/streptomycin (Euroclone, Milan, Italy) and 10% fetal bovine serum (Gibco, Grand Island, NY, USA). Cell viability was measured by the MTT assay. Briefly, the cells were seeded in 96-well plates (5000 cells/well) and exposed for 24 h to *O. illyricum* solution (2 mg/mL in DMSO) or *O. illyricum* liposomes/PG-PEVs, properly diluted to reach the required extract concentrations (0.1, 1, 2.5, 5, and 10 µg/mL). Thereafter, the MTT solution (0.5 mg/mL) was added. After 3 h, DMSO was added to dissolve the formazan crystals, and the absorbance was recorded at 590 nm.

### 4.9. Antioxidant Activity in Cells

The antioxidant activity of *O. illyricum* extract was assessed by analyzing the intracellular ROS levels via the DCFH-DA method. HaCaT cells were incubated with the samples diluted to achieve the required extract concentrations (0.1, 1, 2.5, 5, and 10 µg/mL) for 24 h. Then, the cells were incubated with 10 µM DCFH-DA for 30 min. After the incubation, 2 mM H_2_O_2_ was added to each well, and the fluorescence intensity of ROS-oxidized 2′,7′-dichlorofuorescein (DCF) was measured at 485/530 nm (excitation/emission wavelengths), recording data for 60 min.

### 4.10. Statistical Analysis

The results are reported as means ± standard deviations (SD). Student’s *t*-test was performed to substantiate differences between groups (i.e., the vesicle formulations). For cell viability and intracellular antioxidant activity data, a two-way analysis of variance (ANOVA) was performed using the type of extract (free or nanoformulated) and the different concentrations tested as variables, followed by Tukey’s test, using GraphPad Prism software version 9 (San Diego, CA, USA). Differences were considered statistically significant for *p* values below 0.05.

## 5. Conclusions

The results of this study demonstrate that the antioxidant power of a phenolic-rich extract from *O. illyricum* leaves was enhanced by the incorporation in nanovesicles. This supports the role that nanotechnologies can play to allow and even promote the application of plant-derived products in therapy. Further investigation is certainly required to understand the interactions between extract-loaded vesicles and cells and to assess the penetration of targeted phenolic compounds in the skin.

## Figures and Tables

**Table 1:** Compounds identified by (HR) LC-ESI-QTOF MS/MS in *O. illyricum* extract.

Compound No.	Rt min	Identity	UV λmax nm	[M-H]^−^ *m*/*z*	Molecular Formula	Δ ppm	MS/MS * m/*z*	References	Level #
1	8.96	5-*O*-caffeoylquinic acid	218, 326	353.0884	C_16_H_18_O_9_	0.39	191.0559(100)	[4,19]	1
2	12.16	1,3-Dicaffeoylquinic acid	244, 322	515.1197	C_25_H_24_O_12_	0.18	353.0884(12)/191.0567(100)	[4,19,20]	1
3	13.14	Feruloylquinic acid	244, 328	367.1038	C_17_H_20_O_9_	0.68	193.0482(11)/191.0560(100)	[19]	2
4	17.10	Luteolin glucuronide	256, 267, 348	461.0725	C_21_H_18_O_12_	−0.04	285.0404(100)	[21]	2
5	17.73	3,4-Dicaffeoylquinic acid	244, 328	515.1197	C_25_H_24_O_12_	0.15	353.0883(9)/191.0567(100)	[4,19,20]	1
6	17.99	3,5-Dicaffeoylquinic acid	242, 328	515.1197	C_25_H_24_O_12_	0.26	353.0882(15)/191.0567(100)	[4,19,20]	1
7	18.56	Dicaffeoylquinic acid	242, 328	515.1197	C_25_H_24_O_12_	0.44	353.0863(12)/191.0562(100)/179.0339(29)	[4,19,20]	2
8	18.77	1,5-Dicaffeoylquinic acid	245, 330	515.1197	C_25_H_24_O_12_	0.37	353.0863(7)/191.0562(100)/179.0339(8)	[4,19,20]	2
9	19.32	Apigenin rutinoside	48, 338	577.1564	C_27_H_30_O_14_	0.02	269.0452(100)	[4]	2
10	19.76	Dicaffeoylsuccunoylquinic acid I	243, 330	615.1361	C_29_H_28_O_15_	0.52	515.06924(9)/353.0855(9)/191.0598(98)	[4,9,20]	2
11	19.91	4,5-Dicaffeoylquinic acid	244, 328	515.1197	C_25_H_24_O_12_	−0.06	353.0883(14)/191.0567(100)	[4,19,20]	1
12	20.24	Dicaffeoylsuccunoylquinic acid II	244, 330	615.1361	C_29_H_28_O_15_	0.52	515.0774(20)/353.0821(11)/191.0564(100)	[4,9,20]	2
13	20.73	Hispidulin glucuronide	270, 334	475.0880	C_22_H_20_O_12_	−0.41	299.0535(100)/284.0334(57)	[22]	2
14	22.61	Feruloylcaffeoylquinic acid	244, 330	529.1354	C_26_H_26_O_12_	0.68	367.1038(15)/353.0867(7)/191.0560(100)	[19]	2
15	24.39	Luteolin	256, 268, 346	285.0398	C_15_H_10_O_6_	−0.04	212.0463(25)/171.0460(12)	[4,8]	1
16	30.02	Hispidulin	274, 337	299.0558	C_16_H_12_O_6_	−1.05	284.0334(100)	[4,8]	1

* in parentheses the relative intensity; # according to Blaženović et al. [18].

**Table 2:** Concentration of targeted phenolic compounds detected in *O. illyricum* extract (mg/g extract dry mass, mean ± SD; *n* = 3).

Compound	No.	*O. illyricum* Extract (mg/g dm)
		Mean ± SD
**Total Hydroxycinnamic acids**		**9.59 ± 0.07**
5-*O*-caffeoylquinic acid	1	1.30 ± 0.02
1,3-di-*O*-caffeoylquinic acid	2	0.1 ± 0.00
Feruloyl quinic acid ^A^	3	0.13 ± 0.00
3,4-di-*O*-caffeoylquinic acid	5	0.35 ± 0.00
3,5-di-*O*-caffeoylquinic acid	6	0.44 ± 0.01
Dicaffeoylquinic acid ^B^	7	0.67 ± 0.00
1,5-di-*O*-caffeoylquinic acid ^B^	8	3.40 ± 0.07
Dicaffeoyl succinoyl quinic acid I ^B^	10	0.30 ± 0.01
4,5-di-*O*-caffeoylquinic acid	11	0.77 ± 0.02
Dicaffeoyl succinoyl quinic acid II ^B^	12	1.70 ± 0.00
Feruloyl caffeoyl quinic acid ^A^	14	0.44 ± 0.01
**Total Flavonols**		**7.32 ± 0.05**
Luteolin-glucuronide ^C^	4	4.37 ± 0.00
Apigenin-rutinoside ^D^	9	0.24 ± 0.00
Hispidulin-glucuronide ^E^	13	0.28 ± 0.00
Luteolin	15	1.14 ± 0.01
Hispidulin	16	1.30 ± 0.02
**Total polyphenols**		**16.91 ± 0.83**

^A^ dosed as ferulic acid equivalents; ^B^ dosed as 1,3-di-*O*-caffeoylquinic acid equivalents; ^C^ dosed as luteolin equivalents; ^D^ dosed as apigenin equivalents; ^E^ dosed as hispidulin equivalents.

**Table 3:** Characteristics of empty and *O. illyricum* liposomes and PG-PEVs: mean diameter (MD), polydispersity index (PI), and zeta potential (ZP).

Formulation	MD nm ± SD	PI ± SD	ZP mV ± SD
Empty liposomes	80 ± 3.6	0.31 ± 0.04	−14 ± 1.4
*O. illyricum* liposomes	*** 96 ± 3.3	*** 0.24 ± 0.01	*** −6 ± 1.6
Empty PG-PEVs	77 ± 10.1	0.36 ± 0.08	−15 ± 2.8
*O. illyricum* PG-PEVs	^§§§^ 77 ± 3.1	^§§§,^ °°° 0.22 ± 0.01	°°° −6 ± 1.2

Each value represents the mean ± SD (*n* > 10). *** values statistically different (*p* < 0.001) from empty liposomes; °°° values statistically different (*p* < 0.001) from empty PG-PEVs; ^§§§^ values statistically different (*p* < 0.001) from *O. illyricum* liposomes.

**Table 4:** Mean diameter (MD), polydispersity index (PI), and zeta potential (ZP) of empty and *O. illyricum* liposomes and PG-PEVs after 2 months of storage (t_2_) vs. the data generated from time zero (t_0_).

Formulation	Time	MD nm ± SD	PI ± SD	ZP mV ± SD
Empty liposomes	t_0_	80 ± 3.6	0.31 ± 0.04	−14 ± 1.4
	t_2_	* 91 ± 4.7	0.39 ± 0.08	−15 ± 2.8
*O. illyricum* liposomes	t_0_	96 ± 3.3	0.24 ± 0.01	−6 ± 1.6
	t_2_	103 ± 2.4	^§^ 0.28 ± 0.02	−10 ± 2.2
Empty PG-PEVs	t_0_	77 ± 10.1	0.36 ± 0.08	−15 ± 2.8
	t_2_	90 ± 6.3	0.42 ± 0.07	−17 ± 3.8
*O. illyricum* PG-PEVs	t_0_	77 ± 3.1	0.22 ± 0.01	−6 ± 1.2
	t_2_	84 ± 4.1	0.25 ± 0.03	−10 ± 3.5

Each value represents the mean ± SD (*n* = 4). * value statistically different (*p* < 0.05) from empty liposomes at t_0_; ^§^ value statistically different (*p* < 0.05) from *O. illyricum* liposomes at t_0_.

**Table 5:** Entrapment efficiency (EE%) of the main phenolic compounds identified in *O. illyricum* extract.

Peak No.	Compound	EE% ± SD	
		PG-PEVs	Liposomes
	**Hydroxycinnamic acids**		
1	5-O-caffeoylquinic acid	38.8 ± 5.4 ^a^	60.0 ± 2.5 ^b^
2	1,3-Dicaffeoylquinic acid	43.0 ± 6.7 ^a^	66.7 ± 3.4 ^b^
3	Feruloyl quinic acid ^A^	45.8 ± 4.8 ^a^	57.1 ± 7.8 ^a^
5	3,4-Dicaffeoylquinic acid	97.6 ± 5.0 ^a^	95.6 ± 9.8 ^a^
6	3,5-Dicaffeoylquinic acid	93.5 ± 4.3 ^a^	93.1 ± 6.1 ^a^
7	Dicaffeoylquinic acid ^B^	83.0 ± 5.6 ^a^	96.0 ± 7.8 ^a^
8	1,5-Dicaffeoylquinic acid ^B^	88.2 ± 8.7 ^a^	77.8 ± 8.8 ^a^
10	Dicaffeoylsuccunoylquinic acid I ^B^	82.6 ± 3.5 ^a^	99.3 ± 5.5 ^b^
11	4,5-Dicaffeoylquinic acid	80.7 ± 4.9 ^a^	94.5 ± 4.8 ^b^
12	Dicaffeoylsuccunoylquinic acid II ^B^	77.3 ± 9.8 ^a^	69.6 ± 6.8 ^a^
14	Feruloyl caffeoyl quinic acid ^A^	89.2 ± 7.2 ^a^	100.3 ± 7.2 ^a^
	**Flavonols**		
4	Luteolin glucuronide ^C^	82.5 ± 6.1 ^a^	97.3 ± 2.7 ^b^
13	Hispidulin glucuronide ^D^	77.5 ± 5.1 ^a^	94.9 ± 5.5 ^b^

^A^ Dosed as ferulic acid equivalents. ^B^ Dosed as 1,3-di-*O*-caffeoylquinic acid equivalents. ^C^ Dosed as luteolin equivalents. ^D^ Dosed as hispidulin equivalents. Mean values within a line with different letters are significantly different at *p* ≤ 0.05. Data are given as means ± standard deviations (SD) (*n* = 4).

**Table 6:** In vitro antioxidant activity of *O. illyricum* formulations. For the DPPH assay, results are expressed as AA (antioxidant activity %) and TE (μg Trolox equivalents/mL solution); for the FRAP assay, results are expressed as FE (µg Fe^2+^ equivalents/mL solution).

Formulation	DPPH Assay		FRAP Assay
	AA (%)	TE (µg Trolox Equivalents/mL)	FE (µg Fe^2+^ Equivalents/mL)
*O. illyricum* solution	94 ± 4.4	673 ± 23	445 ± 15
Empty liposomes	39 ± 9.9	262 ± 28	186 ± 9
*O. illyricum* liposomes	99 ± 0.3	715 ± 3	** 590 ± 19
Empty PG-PEVs	39 ± 6.9	265 ± 32	186 ± 15
*O. illyricum* PG-PEVs	99 ± 0.2	716 ± 2	** 560 ± 6

Results are reported as the mean ± SD of at least three separate experiments, each performed in triplicate. ** Values statistically different (*p* < 0.01) from the *O. illyricum* solution.

**Table 7:** Composition of the vesicle formulations.

Formulation	P90G ^1^	*O. illyricum* ^2^	PG ^3^	H_2_O
Empty liposomes	90 mg	--	--	1 mL
*O. Illyricum* liposomes	90 mg	2 mg	--	1 mL
Empty PG-PEVs	90 mg	--	0.1 mL	0.9 mL
*O. illyricum* PG-PEVs	90 mg	2 mg	0.1 mL	0.9 mL

^1^ phospholipid; ^2^ *O. illyricum* extract; ^3^ propylene glycol.

## Data Availability

The data presented in this study are available within this article.
